# High-Resolution Transcriptomic Analyses of *Sinorhizobium* sp. NGR234 Bacteroids in Determinate Nodules of *Vigna unguiculata* and Indeterminate Nodules of *Leucaena leucocephala*

**DOI:** 10.1371/journal.pone.0070531

**Published:** 2013-08-02

**Authors:** Yan Li, Chang Fu Tian, Wen Feng Chen, Lei Wang, Xin Hua Sui, Wen Xin Chen

**Affiliations:** 1 State Key Laboratory of Agrobiotechnology, College of Biological Sciences, China Agricultural University, Beijing, China; 2 Key Laboratory of Soil Microbiology, Ministry of Agriculture, China Agricultural University, Beijing, China; 3 Rhizobium Research Center, China Agricultural University, Beijing, China; National Center for Cell Science, India

## Abstract

The rhizobium-legume symbiosis is a model system for studying mutualistic interactions between bacteria and eukaryotes. *Sinorhizobium* sp. NGR234 is distinguished by its ability to form either indeterminate nodules or determinate nodules with diverse legumes. Here, we presented a high-resolution RNA-seq transcriptomic analysis of NGR234 bacteroids in indeterminate nodules of *Leucaena leucocephala* and determinate nodules of *Vigna unguiculata*. In contrast to exponentially growing free-living bacteria, non-growing bacteroids from both legumes recruited several common cellular functions such as *cbb3* oxidase, thiamine biosynthesis, nitrate reduction pathway (NO-producing), succinate metabolism, PHB (poly-3-hydroxybutyrate) biosynthesis and phosphate/phosphonate transporters. However, different transcription profiles between bacteroids from two legumes were also uncovered for genes involved in the biosynthesis of exopolysaccharides, lipopolysaccharides, T3SS (type three secretion system) and effector proteins, cytochrome *bd* ubiquinol oxidase, PQQ (pyrroloquinoline quinone), cytochrome *c550*, pseudoazurin, biotin, phasins and glycolate oxidase, and in the metabolism of glutamate and phenylalanine. Noteworthy were the distinct expression patterns of genes encoding phasins, which are thought to be involved in regulating the surface/volume ratio of PHB granules. These patterns are in good agreement with the observed granule size difference between bacteroids from *L. leucocephala* and *V. unguiculata*.

## Introduction

The rhizobium-legume symbiosis has been a model system for studying the mutualistic interactions for many years [1]. This system is characterized by its ability to form the symbiotic nodules, in which rhizobia differentiate into bacteroids to fix atmospheric nitrogen to the benefit of the legume while the host provides carbon sources to these microsymbionts. Symbiotic nodules could be simply categorized into either determinate or indeterminate nodules based on the activity of the nodule meristem. In the determinate nodules (such as those of *Lotus japonicus*, *Glycine max* and *Phaseolus vulgaris*), the meristem functions until the formation of the nodule primordium and produces synchronously developed infected cells, whereas the meristem persists in the indeterminate nodules (such as *Medicago sativa*, *Pisum sativum* and *Vicia sativa*) which are composed of different zones showing a clear developmental gradient: the apical meristem, the invasion zone, the interzone, the nitrogen-fixing zone, and the senescence zone [2], [3], [4]. Enlarged and nonreproductive bacteroids were thought to be a characteristic feature of indeterminate nodules of Inverse-Repeat Legume Clade (IRLC) including *M. sativa*, *P. sativum* and *V. sativa*, in contrast, morphologically unchanged and reproductive bacteroids were commonly found in determinate nodules of Milletioids plants including *L. japonicus*, *G. max* and *P. vulgaris* [5]. However, the latest study of the distribution of these two kinds of bacteroids in 40 legume species in the subfamily Papilionoideae [6] suggested that there was no clear correlation between nodule types and morphologies of bacteroids. Moreover, morphologically unchanged bacteroids might be the ancestral form to enlarged bacteroids [6].

High throughput transcriptomics and proteomics studies have revealed global gene expression profiles of enlarged bacteroids in indeterminate nodules [7], [8], [9], [10], [11] or morphologically unchanged bacteroids in determinate nodules [12], [13], [14], [15], [16]. Few of them compared host-specific bacteroid transcripts and/or proteins [11], [16]. Recently, exciting progresses have been made in identifying the determinants of the interactions between host cells and enlarged bacteroids in indeterminate nodules of *Medicago* and *Pisum* [17], [18], [19], [20], [21]. But little information was known for the persistence mechanisms of both types of bacteroids in determinate nodules and morphologically unchanged bacteroids in indeterminate nodules, partially due to different strategies used by diverse legume-rhizobium systems [2], [6], [22], [23]. *Sinorhizobium* sp. NGR234 is well known for its ability to nodulate legume hosts from as many as 112 genera forming either determinate or indeterminate nodules [24]. Thus NGR234 could serve as an excellent model for investigating the adaptation mechanisms of rhizobia to diverse conditions within different types of nodules. In this study, RNA-seq was used to investigate the transcriptomic differences between free-living NGR234 and NGR234 bacteroids in either determinate nodules of *V. unguiculata* or indeterminate nodules of *L. leucocephala*. Both common and distinct transcription patterns of NGR234 bacteroids in these two legumes were analyzed.

## Materials and Methods

### Growth Conditions for Bacterial Strain and Plants

The broad-host range strain *Sinorhizobium* sp. NGR234 [24] was grown in liquid TY (tryptone yeast extract) medium [25] at 28°C. Bacterial culture with OD_600_ = 0.6 (the optical density at 600 nm) was used for inoculating legume hosts *V. unguiculata* and *L. leucocephala* forming determinate and indeterminate nodules respectively with NGR234. Seeds of *V. unguiculata* were surface-sterilized by successive treatments with 95% ethanol for 30 sec and 0.2% (w/v) HgCl_2_ for 5 min, and were then washed for 10 times by using autoclaved deionized water. Seeds of *L. leucocephala* were first treated with sulfuric acid for 30 min, followed by washing six times in sterilized water. They were then surface-sterilized as described for seeds of *V. unguiculata*. All the surface-sterilized seeds were germinated on 0.6% agar–water plates in the dark at 28°C for 24–48 h. Germinated seeds were planted in vermiculite moisturized with low-N nutrient solution in Leonard jars [26] and were inoculated with 1 ml of bacterial culture with OD_600_ = 0.6 per plant. All the plants were grown at 24°C in a plant growth room with a daylight illumination period of 12 h. Nodules were harvested 21 DPI (days post inoculation) for *V. unguiculata* and 35 DPI for *L. leucocephala* when the acetylene reductase activity of nodules reached the peak level. Three biological replications were done. These nodules were either frozen in liquid nitrogen and stored at –80°C until RNA extraction, or used for sample preparations for electron and light microscopy.

### Light and Electron Microscopy

Nodules were fixed in 2.5% glutaraldehyde in 0.05 M cacodylate buffer [27]. For light microscopy, fixed nodules were washed, dehydrated, embedded in Technovit 7100 (Kulzer Histo-Technik), according to the manufacturer’s instructions. Sections of 2 µm were cut on a Leica Ultracut C6i and stained with 1% toluidine blue for 40 sec. For electron microscopy, fixed nodules were washed with 0.1 M phosphate buffer and postfixed in 0.1 M phosphate buffer containing 1% (wt/vol) OsO_4_. The samples were then washed with 0.1 M phosphate buffer and dehydrated with increasing volumes of acetone (30%, 50%, 70%, 90%, and 100%). The samples were embedded in the SPURR epoxy. A Leica Ultracut C6i was used to obtain ultrathin sections (80 nm thick) of these nodule samples. The resulting sections were stained with uranyl acetate and lead citrate and finally observed in JEM-1230 transmission electron microscope.

### Isolation and Purification of Bacteroids

A slightly modified approach described by Day et al. [28] was used for bacteroid isolation from frozen nodules of *V. unguiculata* and *L. leucocephala*. Briefly, 2–5 g nodules were extensively ground by using a pre-chilled pestle in 15 ml of extraction buffer (10 mM DTT, 300 mM sucrose, 10 mM phosphate buffer pH 7.0, 2 mM MgCl_2_ and 0.33 g PVP). In order to remove large particles of plant cell debris, the mixture was centrifuged at 400×g for 10min, 4°C. The supernatant was centrifuged at 12000×g for 10 min, 4°C. The resulting pellet containing bacteroids and small plant cell debris was resuspended in 10 ml extraction buffer. Then 10 ml 30% percoll, 5 ml 60% percoll and 5 ml 80% percoll were added into the same tube. After the centrifugation at 4000×g for 15 min, 4°C, the layer between 60%–80% percoll containing bacteroids was diluted into 20 ml using the extraction buffer. The resulting solution was transferred to the tube containing 5 mL 60% percoll and 5 mL 80% percoll and subject to another round of centrifugation at 4000×g for 15 min, 4°C. The cushion above 80% percoll containing bacteroids was resuspended in 20 mL 0.8% NaCl and centrifuged at 12000×g for 5 min. The pellet was collected for RNA extraction of bacteroids.

### RNA Extraction

Bacteroids were ground by using a pestle in liquid nitrogen. They were then subjected to RNA extraction using QIAGEN RNeasy mini kit according to the manufacturer’s instructions. For free-living bacteria, RNA from a bacterial culture with OD_600_ = 0.5 was extracted by using the same QIAGEN RNeasy mini kit. RNA quality was assessed by using an Agilent 2100 Bioanalyzer. RNA integrity number (RIN, average ± SE) was 8.80±0.10, 9.87±0.13 and 9.93±0.07 for RNA sample of *L. leucocephala* bacteroids, *V. unguiculata* bacteroids and the free culture of NGR234, respectively, indicating the good quality of RNA samples in this study.

### Library Construction and Strand-specific RNA Sequencing

Total RNA was sent to BGI-Shenzhen for further treatments, library construction and strand-specific RNA sequencing. Briefly, total RNA was treated with RNase-free DNase I for 30 min at 37°C to remove residual DNA. Total RNA was then treated with Ribo-Zero rRNA Removal Kit (Gram-Negative Bacteria) according to the manufacturer’s instructions to remove the ribosomal RNA before preparing RNA libraries for deep sequencing. 5 µg of total RNA was used as the starting material for treatment. The mRNA-enriched RNA was chemically fragmented to 150∼200 bp using divalent cations under elevated temperature. The cleaved RNA fragments were copied into first strand cDNA using reverse transcriptase and random primers. Non-incorporated nucleotides were removed and dTTP was substituted by dUTP during the synthesis of the second strand [29]. These cDNA then went through an end repair process, the addition of a single “A” base, and then ligation of adapters. The ligation products were then purified and subsequently digested with N-glycosylase (UNG; Applied Biosystems) to remove the second-strand cDNA. The products were then enriched by 15 cycles of PCR cycles with phusion polymerase to create the final cDNA library. Libraries were sequenced on an Illumina Hiseq 2000 platform.

### Sequence Analyses

Clean reads were mapped to the reference genome of *Sinorhizobium* sp. NGR234 [30] using SOAP2 [31]. Mismatches no more than 5 bases were allowed in the alignment. To eliminate the influence of different gene length and sequencing discrepancy on the calculation of gene expression, the RPKM (reads per kilobase per million mapped reads) method was used to calculate gene expression level [32]. Genes with the ratio of RPKMs of the two samples above 2, Benjamini FDR (False Discovery Rate) ≤0.001 and the coverage value larger than 80% in the transcriptionally up-regulated condition were chosen as the differentially expressed genes (DEGs) between two samples. IGV [33] was used to visualize the expression patterns across the genome. KEGG pathway annotations for *Sinorhizobium* sp. NGR234 were retrieved from the KEGG database [34] and used in the pathway enrichment analysis of DEGs by using Gitools [35]. The Benjamini FDR corrected P value <0.05 (two-tailed) for Fisher exact test was used to define the enriched pathway.

### qRT-PCR

To validate the results of RNA-seq, quantitative reverse transcription PCR (qRT-PCR) experiments were performed in triplicate for 13 genes having different expression profiles. Three biological replicates were analyzed. Single strand cDNA was synthesized by using the GoScript™ Reverse Transcription System kit (Promega). Quantitative PCR was performed by using 25 µL of Light Cycler 480 SYBR Green I Master (Roche) and a Light Cycler 480 real-time PCR system (Roche). The PCR procedures were as follows: 95°C for 2 min; 40 cycles of 95°C for 15 sec, 60°C for 1 min. PCR results were analyzed by relative quantification methods using the 16S rRNA gene (NGR_c26520) as the reference gene.

## Results and Discussion

### Distinct Characteristics of Nodules from *V. unguiculata* and *L. leucocephala*

**Figure 1:**
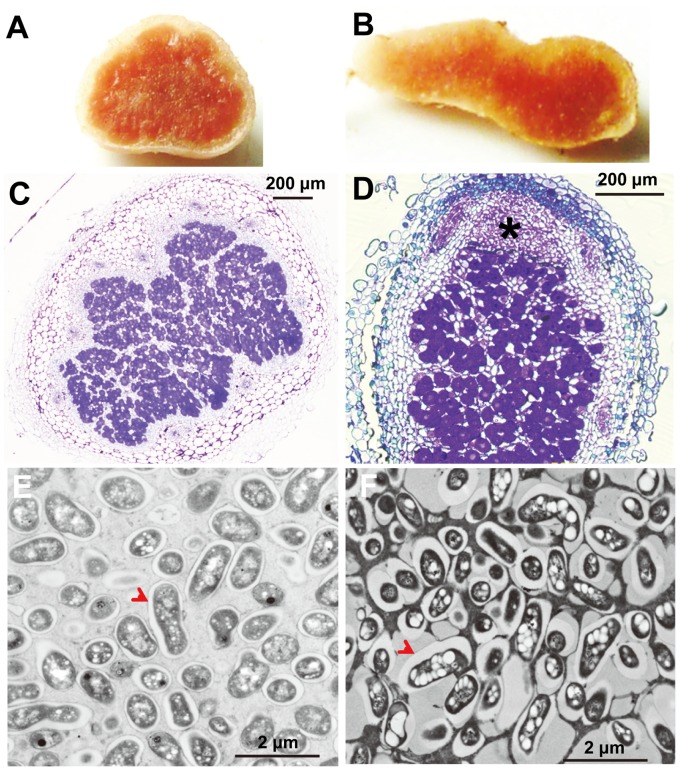
Nodule sections of *Vigna unguiculata* and *Leucaena leucocephala*. (A–B) Nitrogen fixing nodules of *V. unguiculata* (21 days post inoculation, A) and *L. leucocephala* (35 days post inoculation, B). (C–D) Semithin sections of nodules from *V. unguiculata* (C) and *L. leucocephala* (D), the asterisk indicates the meristem zone of indeterminate nodules of *L. leucocephala* (D). (E–F) Ultrathin sections of nodules from *V. unguiculata* (E) and *L. leucocephala* (F), red arrowheads indicate the peribacteroid membrane; and PHB granules are seen as electron-transparent droplets in the cytoplasm of bacteroids.

*Sinorhizobium* sp. NGR234 formed spherical nodules on *V. unguiculata* and elongated nodules on *L. leucocephala* (Figure 1A and 1C). These elongated indeterminate nodules contain a typical meristem zone on the distal part of nodules (Figure 1D). However, bacteroids in determinate or indeterminate nodules of these two legume hosts show several distinct characteristics compared to those in the typical determinate (*L. japonicus*, *G. max* etc.) or indeterminate (*M. truncatula*, *P. sativa* etc.) nodules [2]. Bacteroids in nodules of *V. unguiculata* (Figure 1E) and *L. leucocephala* (Figure 1F) are <2.5 µm in length, suggesting that they all belong to the morphologically unchanged bacteroids [6]. Moreover, there is just one bacteroid surrounded by each peribacteroid membrane in nodules from these two legume hosts (Figure 1E and 1F). Notably, poly-3-hydroxybutyrate (PHB) granules in bacteroids of *L. leucocephala* nodules (Figure 1F) were larger than those in bacteroids of *V. unguiculata* (Figure 1E).

### Gene Expression Overview

RNA-Seq experiments produced 1.169 Gb sequences for the free-living bacteria, *V. unguiculata* and *L. leucocephala* bacteroids, respectively. As shown in Table 1, 97.92%, 87.23% and 56.83% of obtained reads in the three treatments were mapped to *Sinorhizobium* sp. NGR234 genome. These mapping results were consistent with a relatively higher level of the contamination by plant RNA in the RNA samples of *L. leucocephala* bacteroids than in *V. unguiculata* bacteroids (data not shown). However, around 80 Mb sequences from the *L. leucocephala* bacteroids’ sample were uniquely mapped to known CDS of *Sinorhizobium* sp. NGR234 genome (6.9 Mb), and this was among the largest RNA-seq data relative to the genome size of bacteria [36], [37], [38], [39].

**Table 1:** An overview of the RNA-Seq data.

	Free-living bacteria		*V. unguiculata* bacteroids		*L. leucocephala* bacteroids	
	number	%*	number	%*	number	%*
**Total reads^#^**	12988910		12988910		12992456	
**Reads mapped to genome**	12718213		11329803		7383865	
perfect match	9760662	76.75%	8638335	76.24%	5642244	76.41%
< = 5 bp mismatch	2957551	23.25%	2691468	23.76%	1741621	23.59%
reads mapped to rRNA	1082203	8.51%	2519658	22.24%	2847571	38.56%
**Reads mapped to gene**	7705821		3931113		1049762	
perfect match	5920834	76.84%	3022804	76.89%	811630	77.32%
< = 5 bp mismatch	1784987	23.16%	908309	23.11%	238132	22.68%
unique match	7629003	99.00%	3390818	86.26%	886105	84.41%

Note: #, 90-base reads; *, the percentage value relative to the reads mapped to genome or gene.

**Figure 2:**
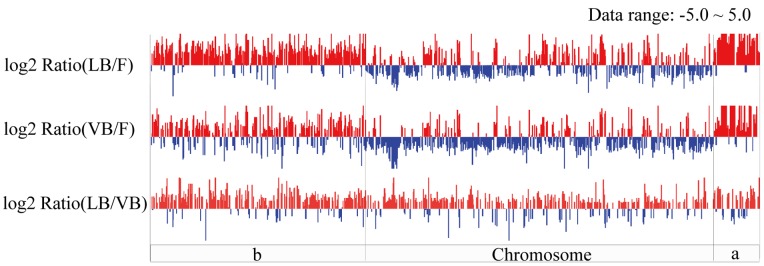
Distributions of differentially expressed genes across replicons of *Sinorhizobium* sp. NGR234. a, pNGR234a; b, pNGR234b; Chromosome, cNGR234; three replicons were separated by dashed lines. log2 Ratio(VB/F), the base 2 logarithm value for the ratio of expression level between bacteroids of *Vigna unguiculata* (VB) and free-living (F) *Sinorhizobium* sp. NGR234; LB/F, the ratio between *L. leucocephala* bacteroids and free-living bacteria; LB/VB, the ratio between *L. leucocephala* bacteroids and *V. unguiculata* bacteroids. The red bars above the horizontal line represent genes with the log2 Ratio values >1.0 whereas the blue bars below the horizontal line indicate genes with the log2 Ratio values<−1.0.

Transcriptomes of bacteroids from nodules of *V. unguiculata* (21 DPI) and *L. leucocephala* (35 DPI) were compared to that of free-living *Sinorhizobium* sp. NGR234 in exponential growth stage (Table S1). As shown in Table 2 and Table S2, among the 3143 DEGs between *V. unguiculata* bacteroids and free-living bacteria, 90.2% DEGs of pNGR234a, 66.4% DEGs of pNGR234b and 29.1% DEGs of the chromosome were up-regulated in *V. unguiculata* bacteroids. Similarly, 96.6% DEGs of pNGR234a, 84.1% DEGs of pNGR234b and 35.9% DEGs of the chromosome were up-regulated in *L. leucocephala* bacteroids compared to those of free-living bacteria (Table 2 and Table S2). These biased distribution patterns of DEGs in three replicons of *Sinorhizobium* sp. NGR234 (Figure 2) also suggested an active role of two plasmids in symbiotic adaptations to both determinate (*V. unguiculata*) and indeterminate (*L. leucocephala*) nodules. This is consistent with the view that rhizobial extrachromosomal elements are important in niche adaptations [40]. Moreover, *V. unguiculata* and *L. leucocephala* bacteroids shared a common subset of 2072 DEGs compared to free-living bacteria (Table S2), and 99.1% of these DEGs showed the same direction of regulation in both legumes hosts. Moreover, among these 2072 DEGs, 141/146 DEGs of pNGR234a, 391/523 DEGs of pNGR234b and 412/1403 DEGs of the chromosome were up-regulated in both *V. unguiculata* and *L. leucocephala* bacteroids. As shown in Table S3, the enrichment analysis revealed that up-regulated DEGs in bacteroids from both hosts were particularly enriched in the KEGG pathways for microbial metabolism in diverse environments (rhi01120), ABC transporters (rhi02010), nitrogen metabolism (rhi00910), fatty acid metabolism (rhi00071) and benzoate degradation (rhi00362). In contrast, down-regulated genes were enriched in the KEGG pathways for ribosomes (rhi03010), pyrimidine metabolism (rhi00240), flagellar assembly (rhi02040) and aminoacyl-tRNA biosynthesis (rhi00970). Despite these similar expression profiles, distinct transcriptional differences were also observed between bacteroids from *V. unguiculata* and *L. leucocephala* such as phenylalanine metabolism (rhi00360) (Table S2-S3 and see discussion below).

**Table 2:** Differentially expressed genes in three replicons of *Sinorhizobium* sp. NGR234.

Comparisons	DEG	pNGR234a	pNGR234b	Chromosome
VB/F	3143	166∶18*	589∶298	603∶1469
LB/F	2780	201∶7	747∶141	604∶1080
LB/VB	1184	76∶28	428∶78	424∶150

Note: VB/F, *V. unguiculata* bacteroids vs free-living bacteria; LB/F, *L. leucocephala* bacteroids vs free-living bacteria; LB/VB, *L. leucocephala* bacteroids vs *V. unguiculata* bacteroids; DEG, differentially expressed genes in each comparison.

* , in each replicon, the number of genes up-regulated: down-regulated in the corresponding condition.

**Figure 3:**
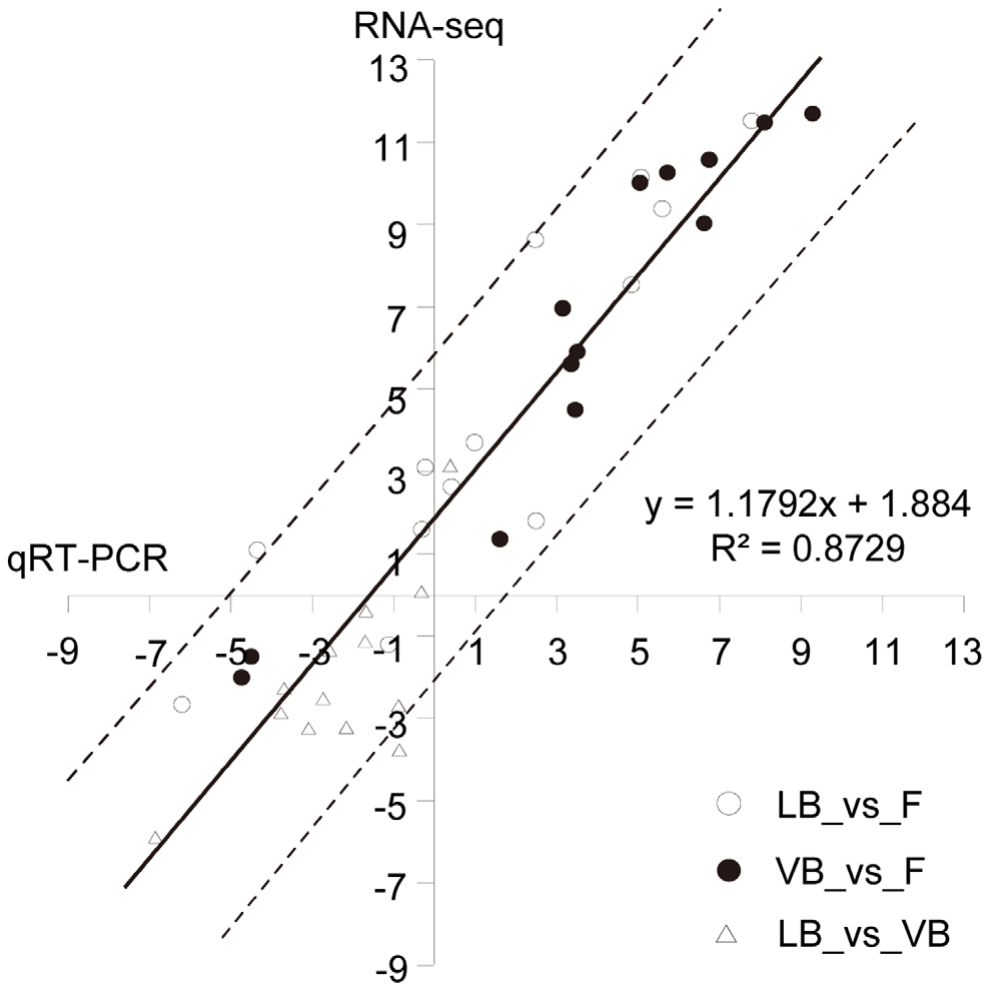
Validation of RNA-seq results by quantitative reverse transcription-polymerase chain reaction (qRT-PCR). Expression of 13 genes was determined using qRT-PCR for the free-living *Sinorhizobium* sp. NGR234, *L. leucocephala* and *V. unguiculata* bacteroids. The expression ratios were calculated for *L. leucocephala* bacteroids vs. free-living (LB_vs_F), *V. unguiculata* bacteroids vs. free-living (VB_vs_F), and *L. leucocephala* bacteroids vs. *V. unguiculata* bacteroids (LB_vs_VB). The log2-transformed expression ratios from RNA-seq (vertical axis) and qRT-PCR (horizontal axis) are shown. The solid and dashed line(s) depicted the linear curve and the individual confidence intervals (95%). The linear equation and R square values are shown.

To validate the results of RNA-seq, we performed qRT-PCR on 13 genes with different expression profiles (Table S4). These include genes up-regulated or down-regulated in both *L. leucocephala* and *V. unguiculata* bacteroids with (or without) significant differences between expression levels in two hosts, and genes reversely regulated in bacteroids from *L. leucocephala* and *V. unguiculata*. As shown in Figure 3, the RNA-seq data agree well with the qRT-PCR data and Pearson correlation coefficient value was 0.934 (*P*<0.0001), despite the few differences that are often observed between qRT-PCR and microarrays results [10] [11] [41]. Similar results were obtained for qRT-PCR experiments with three independent biological replications (Table S4).

### Growth Arrest of Bacteroids in Nodule Environments of *V. unguiculata* and *L. leucocephala*

Consistent with the non-growing state of nitrogen-fixing bacteroids, the Fisher exact test with Benjamini FDR corrected P values (two-tailed) revealed that the down-regulated genes in both *L. leucocephala* and *V. unguiculata* bacteroids (Table S3) were significantly (P<0.05) enriched in pathways for ribosomes (rhi03010), flagellar assembly (rhi02040), aminoacyl-tRNA biosynthesis (rhi00970) and pyrimidine metabolism (rhi00240) among others. Notably, all the flagellar assembly proteins except NGR_c28210 (the second copy of MotB) were down-regulated in bacteroids. This is in contrast to the induction of flagellar proteins in *Bradyrhizobium japonicum* by genistein application and the requirement of flagella in biofilm formation and competitive nodulation of *S. meliloti* [42], [43], [44], [45]. However, it was also reported that nonflagellated mutants of *S. meliloti* formed normal nitrogen-fixing nodules on alfalfa [46], suggesting that flagella are dispensable for function of bacteroids.

Growth arrest of bacteroids was also illustrated by the down-regulation of genes encoding DNA replication proteins (DNA polymerase I, DNA polymerase III subunits chi, DnaE, HolA and DnaQ, replicative DNA helicase, ribonuclease H), homologous recombination proteins (RecAR, Holliday junction DNA helicase RuvAB), chromosome partitioning proteins (ParABC), cell division proteins (FtsAIK_1_K_2_QZ_1_), peptidoglycan biosynthesis proteins (MurABCDEG, MraY, MviN, PbpB, D-alanyl-D-alanine carboxypeptidase NGR_c16080, D-alanine–D-alanine ligase NGR_c21040), core subunits of RNA polymerase (RpoABC), translation factors (initiation factors InfABC, elongation factors Ts, Tu, G and P, peptide chain release factors 1–3, ribosome recycling factor, release factor glutamine methyltransferase) and ATP synthase proteins (AtpABCDF_1_F_2_GH). A similar down-regulation of growth-associated processes was observed in bacteroids of *S. meliloti*, *R. leguminosarum* and *Rhizobium etli* [10], [11], [41].

### ABC Transporters

Among the DEGs between bacteroids and the free-living form, up-regulated genes in bacteroids were particularly enriched in ABC transporters (Table S3 and Table 3). The strong up-regulation of transporters for phosphate (*NGR_c01440-NGR_c01470*, 7–18 folds, RPKM = 314–5089) in bacteroids is consistent with an earlier observation that a phosphate transporter mutant showed a deficiency in nitrogen-fixation [47]. Interestingly, transcriptions of genes coding phosphonate transporters (*NGR_b13310-NGR_b13340*, 58–436 folds, RPKM = 87–1576) and *sn*-glycerol 3-phosphate transporters (*NGR_c36780-NGR_c36810*, 50–428 folds, RPKM = 201–1771) were also strongly induced in bacteroids. These data suggest a phosphorus-limiting nodule environment for bacteroids. Under phosphorus-limiting conditions, *S. meliloti* could replace most of its phospholipids with membrane-forming lipids lacking phosphorus such as DGTS (diacylglyceryl-N,N,N-trimethylhomoserine) [48]. In NGR234 bacteroids from two test hosts, *btaA* and *btaB* (*NGR_c21260- NGR_c21270*, 47–123 folds) involved in the biosynthesis of DGTS were strongly up-regulated, implying that NGR234, regardless of test hosts, could form membrane lipids lacking phosphorus to adapt to the nodule environment. But available studies on *S. meliloti* suggested that phosphorus-free membrane lipids were not required for the symbiosis with alfalfa [49].

**Table 3:** Differentially expressed genes encoding ABC transporters.

Substrate	Gene ID	RPKM_F		RPKM_LB	RPKM_VB	
	Up-regulated in bacteroids from both legume hosts					
phosphate	NGR_c01440-NGR_c01470	42–323		314–4710	670–5089	
phosphonate	NGR_b13310-NGR_b13340	1–4		87–1576	116–1494	
sn-glycerol 3-phosphate	NGR_c36780-NGR_c36810	1–4		150–1711	267–1771	
aminoethane sulfonate	NGR_b17610-NGR_b17630	1–5		37–80	16–31	
aliphatic sulfonate	NGR_c25620-NGR_c25630, NGR_c25650	2–11		170–283	74–153	
osmoprotectants	NGR_b12990-NGR_b13020	5–24		31–312	19–185	
polar amino acids	NGR_b03480- NGR_b03510	1–26		13–157	14–439	
spermidine/putrescine	NGR_b12810-NGR_b12840	1–3		26–149	11–80	
iron (III)	NGR_c19530- NGR_c19550	1–6		56–377	56–369	
simple sugar	NGR_b03950, NGR_b03970-NGR_b03990	2–4		35–169	17–68	
multiple sugar	NGR_b02160-NGR_b02190; NGR_c06670-NGR_c06700;	4–20		18–141	13–191	
	NGR_b04010-NGR_b04030, NGR_b04050;	7–69		30–221	26–515	
	NGR_b20230-NGR_b20260	4–14		28–71	26–176	
	**Down-regulated in bacteroids from both legume hosts**					
alpha-glucoside	NGR_c03100-NGR_c03140	825–2951		35–305	12–256	
trehalose/maltose	NGR_b23110-NGR_b23140	62–364		16–37	1–12	
D-xylose	NGR_c24220-NGR_c24240	477–2200		20–28	6–15	
fructose	NGR_c01100-NGR_c01120	517–3865		42–436	52–949	
dipeptide	NGR_c03460-NGR_c03500	518–2642		56–1055	56–768	
lipoprotein-releasing	NGR_c10690-NGR_c10700	194–328		90–132	77–128	
lipopolysaccharide	NGR_c00180,NGR_c09020-NGR_c09030	165–342		66–87	44–78	
glycine betaine/proline	NGR_c27400- NGR_c27420	94–250		28–37	9–42	
multiple sugar	NGR_c33060- NGR_c33090; NGR_c30950-NGR_c30980	58–455		12–36	3–41	
simple sugar	NGR_c14510-NGR_c14540	97–854		24–356	19–296	
branched-chain amino acids	NGR_c25150, NGR_c25170-NGR_c25200	961–4353		105–1230	189–1296	
peptides/nickel	NGR_c24410-NGR_c24450	66–370		25–108	21–95	
iron complex	NGR_b02510-NGR_b02530; NGR_b11200-NGR_b11230	492–1936		31–38	69–260	

Note: RPKM (reads per kilobase per million mapped reads); F, free-living bacteria; LB, *L. leucocephala* bacteroids; VB, *V. unguiculata* bacteroids.

The *ssu* genes (*NGR_c25620- NGR_c25650*, 10–133 folds) encoding a sulfonate monooxygenase (*ssuD*) and ABC transport proteins (*ssuABC*) for aliphatic sulfonate, and *tauABC* (*NGR_b17610-NGR_b17630*, 6–35 folds) encoding ABC transporters for aminoethane sulfonate were up-regulated in bacteroids. This is consistent with the utilization of aminoethane sulfonate as a sulfur source by *S. meliloti* and an increased expression of putative sulfonate monooxygenases in bacteroids of *G. max* and *Sesbania rostrata* [14] [50].

Transporter proteins of spermidine/putrescine (*NGR_b12810-NGR_b12840*) were specifically expressed in bacteroids (RPKM = 11–149 versus RPKM = 1–3 in the culture condition). This indicated bacterial uptake of these biogenic amines from plants [51].

Although transcription levels of certain transporter proteins for branched-chain amino acids (*NGR_c25150, NGR_c25170-NGR_c25200*, 3–11 folds) were down-regulated in bacteroids, their RPKM values were still at a relatively high level (105–1296). On the other hand, certain transporter proteins for polar amino acids (NGR_b03480- NGR_b03510) expressed higher in bacteroids (RPKM = 13–439) compared to the free-living form (RPKM = 1–26). Interestingly, two transporter proteins for branched-chain amino acids (NGR_c08790- NGR_c08800) were specifically up-regulated in *V. unguiculata* bacteroids (RPKM = 954–1030) compared to *L. leucocephala* bacteroids (RPKM = 39–48) and the free-living form (RPKM = 162–219).

Iron complex coding genes (*NGR_b02510-NGR_b02530*; *NGR_b11200-NGR_b11230*) were highly expressed in the free-living condition (RPKM = 322–1950 versus RPKM = 10–260 in bacteroids), whereas transcriptional levels of ferric ion transporter proteins (*NGR_c19530- NGR_c19550*, 35–122 folds) were specifically induced in bacteroids (RPKM = 56–377 versus RPKM = 1–6 in free-living condition). Certain ABC transporter proteins of osmoprotectants (*NGR_b12990-NGR_b13020*) were induced in bacteroids (RPKM = 19–312 versus RPKM = 5–24 in the free-living condition), while others (*NGR_c27400- NGR_c27420*) were repressed (RPKM = 9–42 versus RPKM = 94–250 in the free-living condition). Similar phenomena were observed for transporter proteins of simple and multiple sugars (Table 3). Moreover, a number of ABC transporters were significantly repressed in bacteroids such as transporter proteins of alpha-glucoside, trehalose/maltose, D-xylose, fructose, dipeptide, lipoprotein-releasing, lipopolysaccharide and peptides/nickel (Table 3). These expression patterns revealed a drastic transcriptional change of ABC transporters between bacteroids and free-living bacteria, suggesting the importance of compound exchange between two symbiotic partners. The observation of overrepresented DEGs in ABC transporters was also documented earlier for other rhizobia-legume symbioses [52].

### Secretion Systems

A remarkable number of secretion systems were found in NGR234 [30]. Among the six Type I transporter genes, *tolC* (*NGR_c13520*) and *prsDE* (*aprDE*, *NGR_b10690* and *NGR_b10700*) were down-regulated in bacteroids (2–3 folds), whereas *NGR_c30050*, *NGR_c30060* and *NGR_c30070* were up-regulated in bacteroids (5.5–16.3 folds). Although the *prsD* mutant of *R. leguminosarum* was defective in nitrogen fixation on peas [53], the role of the Type I secretion system in NGR234 remains elusive.

As to the Type II-linked protein secretion systems, the *gsp* cluster encoding general secretion pathway has restricted phyletic distribution among rhizobial species [30]. Our data provided evidence that this *gsp* cluster (*NGR_c22980- NGR_c23100*) was actively transcribed in the free-living condition (RPKM = 15–109) and showed a reduced expression level in bacteroids (RPKM = 0–34). The conserved twin arginine translocase (TAT) pathway is encoded by *tatABC* (*NGR_c13710-NGR_c13730*) which showed a constitutively high expression level (RPKM = 161–1194) in both free-living and bacteroids. The Sec-SRP (signal recognition particle) system includes inner-membrane proteins (SecD_1_D_2_EY, YajC and YidC), ATPase (SecA), SRP receptor (FtsY), targeting proteins (SecB and Ffh), and signal peptidase I. The expression levels of related coding genes (*NGR_c26720 secA, NGR_c33550 secB, NGR_c02010 secD1, NGR_c13810 secD2, NGR_c11760 secE, NGR_c12100 secY, NGR_c13800 yajC, NGR_c00820 yidC, NGR_c32250 ffh*, *NGR_c08280* coding signal peptidase I) in the free-living condition were 2- to 10-fold of those in bacteroids.

NGR234 has two Type III clusters, and only T3SS-I locus (pNGR234a) was reported to modulate the nodulation of many legume hosts excluding *L. leucocephala* and *V. unguiculata* [30], [54]. However, it was reported that all of the T3SS-I locus genes were induced by flavonoids and that *nolB*, *rhcJ*, *nolU* and *nolV* were detected in mature nodules of *V. unguiculata* [55]. In this study, 13/20 and 19/20 T3SS-I locus genes (*NGR_a00520- NGR_a00700, NGR_a00790*) were up-regulated in *V. unguiculata* bacteroids (2- to 11-fold) and in *L. leucocephala* bacteroids (6- to 172-fold), respectively, compared to the free-living condition. In line with differential expression patterns of T3SS-I locus genes in two hosts, *nopP* (*NGR_a00570*), *nopX* (*NGR_a00700*) and *nopL* (*NGR_a00770*) encoding effector proteins were specifically up-regulated in *L. leucocephala* nodules (RPKM = 273–476 versus RPKM = 13–27 in *V. unguiculata* nodules). Considering the presence of bacteria from infection zone of *L. leucocephala* nodules, our finding is to a certain extent consistent with the view that the stimulation of T3SS coincides with development of the infection thread [56]. Although 8/22 and 22/22 T3SS-II locus genes (pNGR234b, *NGR_b22800- NGR_b23010*) were up-regulated in *V. unguiculata* bacteroids (2–356 folds) and in *L. leucocephala* bacteroids (2–132 folds) respectively, the deletion of seven T3SS-II locus genes (*NGR_b22890*-*NGR_b22950*) up-regulated in both *V. unguiculata* and *L. leucocephala* did not show any defects in symbiosis [30]. The Type IV cluster (*NGR_b10250-NGR_b10360*) was constitutively expressed, but at a very low level in both the free-living condition and nodules (RPKM = 1–40).

### Surface Polysaccharides

An *exo* cluster of genes are involved in the synthesis of exopolysaccharides (EPS) in NGR234 [57]. In this study, *exoKLAMONP* (*NGR_b18340- NGR_b18400*, 2- to 8-fold), *exoXU* (*NGR_b18280- NGR_b18290*, 5- and 3- fold) and *exoYFZ* (*NGR_b18270*, *NGR_b18260*, *NGR_b18240*, 2- to 7-fold) were up-regulated in *L. leucocephala* bacteroids compared to the free-living form, whereas only *exoY* (NGR_b18270, 3-fold), *exoX* (*NGR_b18280*, 3-fold) and *exoN* (*NGR_b18390*, 3-fold) were up-regulated in *V. unguiculata* bacteroids. In line with these expression profiles, functional analyses have revealed that mutants of certain *exo* genes (*exoY, exoF, exoQ, exoK, exoL, exoP*) formed uninfected Fix^-^ nodules on *L. leucocephala* but normal Fix^+^ nodules on *V. unguiculata* [57].

In the free-living culture, NGR234 synthesizes primarily rough lipopolysaccharides (LPS) and only trace amounts of smooth LPS [58]. A new smooth LPS species with a modified lipid A-Core and rhamnan O-antigen is induced by flavonoid and is present in bacteroids of *V. unguiculata* [58], [59], [60]. In line with these observations, putative O-antigen biosynthesis protein coding genes *NGR_b11970* and *NGR_b14100* involved in lipid A biosynthesis were up-regulated in bacteroids of both legumes (2- to 7-fold). Noteworthy the *rgpF-rmlB* gene cluster (*NGR_a03500*-*NGR_a03580*), necessary for the synthesis of rhamnan O-antigen [58], [59], [61], was expressed higher in bacteroids of *L. leucocephala* than in *V. unguiculata* bacteroids and in the free-living condition. It has been reported that this gene cluster is up-regulated in the following signaling pathways: NodD1→SyrM2→NodD2→FixF, and NodD1→TtsI→RmlB-WbgA [62], [63]. These regulation patterns led to the hypothesis that the genes absolutely required for rhamnan production are expressed after the bacteria have entered the plant but before they are released into cortical cells of the nodules [61]. Thus, the higher expression of *rgpF-rmlB* in *L. leucocephala* bacteroids might be due to the mixture of bacteroids with those bacterial cells in infection threads, a distinct characteristic for indeterminate nodules. In contrast to these up-regulated genes involved in the biosynthesis of lipid A and O-antigen in nodules from either *L. leucocephala* or both legumes, genes associated to the core region biosynthesis, *lpsB* and *kdtA* (*NGR_c04250, NGR_c15710*), the synthesis and modification of lipid A, *lpxABD*, *acpXL* (*NGR_c18080, NGR_c13420, NGR_c13440, NGR_c13460, NGR_c18030*), unusual sugar (*NGR_c12790, NGR_c35510, NGR_c04250*), and LPS ABC transporters (*NGR_c00180, NGR_c09020-NGR_c09030*) were down-regulated in bacteroids from both legumes (2- to 11-fold). This suggested a general down-regulation for LPS production in bacteroids. Similarly, *lpxD* (*NGR_c13420* homolog) involved in lipid A synthesis was down-regulated in *R. etli* bacteroids [41]. However, mutants in either *acpXL* down-regulated in bacteroids or members of the *rgpF-rmlB* cluster could not form pink nodules with *V. unguiculata* but efficiently nodulate on *L. leucocephala* [61], [64].

These findings suggested that differences in nodule structures and associated characteristics between *L. leucocephala* and *V. unguiculata* may lead to the observed differential expression patterns of EPS and LPS biosynthesis genes. However, the symbiotic significance of these expression profiles, such as the observed expression of *rgpF-rmlB* cluster in *L. leucocephala*, may depend on their interactions with other cellular functions of bacteria and host responses.

### Energy Metabolism

As expected, in microaerobic environment of nodules from both *V. unguiculata* and *L. leucocephala*, the *nif* genes directly involved in nitrogen fixation were strongly up-regulated. Symbiotic nitrogen fixation is a highly energy-demanding process [65]. In NGR234, there are two cluster of genes *NGR_c22030-NGR_c22190* and *NGR_c10480-NGR_c10630* encoding NADH dehydrogenase, and the former cluster was up-regulated in bacteroids while the latter was down-regulated. However, expression levels of the former genes (RPKM = 10–99) were quite low compared to the latter genes (RPKM = 150–707) in bacteroids. F-type H^+^-transporting ATPase coding genes (*NGR_c31100-NGR_c31140* and *NGR_c04470-NGR_c04500*) were all down-regulated (2- to 6-fold). Both *NGR_c25510-NGR_c25550* and *NGR_c05230-NGR_c05300* encode cytochrome *c* oxidases, the former was up-regulated (11- to 41-fold, RPKM = 27–408) while the latter down-regulated (5- to 27-fold, RPKM = 15–249) in bacteroids. In NGR234, there are two clusters of genes encoding *cbb3* oxidases, locus-I (*NGR_c17970-NGR_c17990*) and locus-II (*NGR_c25780-NGR_c25810*). Both loci were up-regulated in bacteroids, but showing a huge difference in expression levels: RPKM = 2453–3677 for locus-I and RPKM = 26–81 for locus-II. Interestingly, *NGR_c01900* and *NGR_c01910* encoding subunits of cytochrome *bd* ubiquinol oxidase were up-regulated in *L. leucocephala* (3- and 5-fold, respectively) but down-regulated in *V. unguiculata* (3- and 10-fold, respectively). However, it should be noted that expression levels of *NGR_c01900* and *NGR_c01910* in *L. leucocephala* were lower (RPKM = 49–51) than *cbb3* oxidase locus-I (RPKM = 2453–3677). Although it was reported that *Azorhizobium caulinodans* uses both cytochrome *cbb3* and *bd* as terminal oxidases for symbiotic nitrogen fixation [66], *cbb3* oxidase could be the major terminal oxidase for symbiotic nitrogen fixation in bacteroids of both *L. leucocephala* and *V. unguiculata*. It has been reported that the constitutive expression of the thiamine biosynthetic pathway caused the production of *cbb3* oxidase in the free-living condition and an increased capacity in nitrogen fixation during symbiosis [67], [68]. In this study we presented additional evidence that *thiCOGE* (*NGR_b02900-NGR_b02930*) and *thiD* (*NGR_b18410*) were up-regulated (2- to 13-fold) in bacteroids from nodules of both *L. leucocephala* (RPKM = 26 - 46) and *V. unguiculata* (RPKM = 44 -202), with a higher expression level in *V. unguiculata* bacteroids. This finding further supports the view that the thiamine biosynthesis genes are commonly expressed extrachromosomal genes in association with plants [40].

*NGR_b03260- NGR_b03300* involved in pyrroloquinoline quinone (PQQ) biosynthesis were specifically activated in *L. leucocephala* bacteroids (RPKM = 169–580 versus RPKM = 4–19 in *V. unguiculata* bacteroids and the free-living form). PQQ has been found as a redox cofactor for membrane-bound dehydrogenases [69]. The presence of PQQ-dependent glucose dehydrogenase was also reported in rhizobia such as *Rhizobium tropici* and *S. meliloti* etc. [70]. The PQQ-linked glucose dehydrogenase has also been demonstrated as a requirement by *S. meliloti* for optimal nodulation efficiency and competitiveness on alfalfa roots [71]. Close to these *pqq* genes in NGR234 genome, *NGR_b03250* encoding a periplasmic alcohol dehydrogenase with ferricytochrome *c* as the acceptor was specifically up-regulated in *L. leucocephala* bacteroids (RPKM = 1367 versus RPKM = 20 in *V. unguiculata* bacteroids and RPKM = 29 in the free culture). Moreover, *NGR_b03210-NGR_b03240* within the same loci showed a similar expression trend as *NGR_b03250- NGR_b03300*, suggesting their potential role in adapting to the nodule environment of *L. leucocephala*. Proteins encoded by *NGR_b03210-NGR_b03240* include a pseudoazurin, a signal transduction histidine kinase, a FIST containing signal transduction protein [72] and a LuxR family response transcriptional regulator. It would be interesting to study whether these signal transduction systems could regulate the expression of PQQ genes. It has also been shown that PQQ could work as an antioxidant protecting bacteria from oxidative damage, or as a nutrient to support bacterial growth [69]. However, the role of PQQ in rhizobium-legume symbiotic interactions remains largely unknown.

A cluster of genes (*NGR_c14380-NGR_c14420*) were expressed higher in *V. unguiculata* bacteroids (RPKM = 546–2522) than in *L. leucocephala* bacteroids (RPKM = 56–305) and the free-living form (RPKM = 9–20). These include genes encoding glutamine amidotransferase-like protein GlxB, putative glutamate synthase subunits GlxCD and glutamine synthetase GlnT. This is in contrast to the relatively low transcription level of *glt* genes (RPKM = 47–277) encoding glutamine synthetase, and *NGR_c32060* (RPKM = 107–113) encoding glutamine amidotransferase in bacteroids from both hosts. Thus, in addition to the up-regulated glutamine synthetase GlnII (*NGR_b20670*, RPKM = 1807–3916 in *V. unguiculata* and *L. leucocephala* bacteroids versus RPKM = 281 in the free culture), NGR234 actively recruited another set of genes in glutamate metabolism in *V. unguiculata* nodules.

### Role of Nitric Oxide

Recently, nitric oxide (NO) has been detected at different steps of the symbiosis between legumes and rhizobia [73], [74], [75]. Modulation of NO levels was demonstrated to be involved in the establishment and persistence of the symbiosis [73], [76], [77]. In this study, the up-regulation of NapABC (nitrate reductase, *NGR_c10020-NGR_c10040*, 10- to 15-fold) and NirK (nitrite reductase, *NGR_c09950*, 37- to 56-fold), and the down-regulation of NorC (nitric oxide reductase, *NGR_c09850*, 2- to 3-fold) suggested that bacteroids contributed to the NO pool within nitrogen-fixing nodules from both *L. leucocephala* and *V. unguiculata*. In line with this finding, in *M. truncatula* nodules formed by *napA* or *nirK* mutant of *S. meliloti*, the production of NO was decreased by about 35% compared with that of the wild-type control [78]. It was reported that cytochrome *c550* is required for the succinate-dependent nitrite reduction and might be involved in electron transfer to the copper-containing nitrite reductase of *B. japonicum* [79]. In this study, *NGR_b03130* encoding cytochrome *c550* was specifically up-regulated in *L. leucocephala* nodules (RPKM = 186 versus RPKM = 5–6 in *V. unguiculata* nodules and in the free-living condition). In line with this, *NGR_b03210*, encoding a pseudoazurin which was demonstrated to be an electron donor to the copper-containing nitrite reductase in other denitrifying bacteria [80], was also strongly up-regulated in *L. leucocephala* nodules (RPKM = 449) compared to *V. unguiculata* nodules (RPKM = 20) and the free-living condition (RPKM = 11). Although cytochrome *c550* is not required by *B. japonicum* for nitrogen-fixation in determinate nodules of *G. max* [81], potential roles of cytochrome *c550* and/or pseudoazurin in indeterminate nodules such as *L. leucocephala* nodules are still unknown.

### Carbon Metabolism

In bacteroids of nodules from both *L. leucocephala* and *V. unguiculata*, strongly up-regulated genes compared to free-living bacteria also included those encoding the C4-dicarboxylate transporter, DctA (*NGR_b21870*, 331- and 460-fold, respectively), the phosphoenolpyruvate carboxykinase, PckA (*NGR_c33940*, 182- and 219-fold, respectively), and a fructose-bisphosphate aldolase (*NGR_c28100*, 89- and 71-fold, respectively), the acetate kinase AckA (*NGR_b13640*, 118- and 182-fold, respectively) and the phosphate acetyltransferase Pta (*NGR_b13630*, 166- and 228-fold, respectively). These data are consistent with the requirement for succinate metabolism and acetyl-CoA [82], [83], suggesting that both *L. leucocephala* and *V. unguiculata* provide succinate to bacteroids as a carbon source.

In the comparison between *L. leucocephala* bacteroids and *V. unguiculata* bacteroids, DEGs were enriched in the phenylalanine metabolism pathway (rhi00360, Table S3). *paa* genes (*NGR_c26090-NGR_c26200*) were strongly up-regulated in *V. unguiculata* bacteroids (RPKM = 78–1195) compared to *L. leucocephala* bacteroids (RPKM = 12–157) and the free-living culture (RPKM = 7–73). Moreover, *NGR_b02860*, encoding a malonyl-CoA decarboxylase which catalyses the reaction of malonyl-CoA to acetyl-CoA and CO_2_, was also specifically up-regulated in *V. unguiculata* bacteroids. This may lead to a higher yield of acetyl-CoA in *V. unguiculata* bacteroids. *NGR_c30010* (4-hydroxyphenylpyruvate dioxygenase), *NGR_c29980* (homogentisate 1,2-dioxygenase) and *NGR_c29960* (fumarylacetoacetate hydrolase) were expressed higher in *V. unguiculata* bacteroids (RPKM = 358–3133) than in *L. leucocephala* bacteroids (RPKM = 60–303) and the free-living form (RPKM = 75–360). Enzymes encoded by these genes are involved in the production of acetoacetate and fumarate. Therefore, the citrate cycle seems to be more active in *V. unguiculata* bacteroids than in *L. leucocephala* bacteroids.

A considerable number of DEGs encoding enzymes that participate in glyoxylate and dicarboxylate metabolism were also found (Table S3), suggesting a potential role of C2 metabolism in symbiosis. Recently, it was reported that this glyoxylate cycle of *R. leguminosarum* was more strongly induced in the rhizosphere of pea, the compatible legume host of this bacterium, than in that of alfalfa and sugar beet [82]. Moreover, the coincident expressions of glyoxylate cycle genes and a subset of *nif* genes in the chemoautotrophic culture of *B. japonicum* [84] imply potential coordination in transcriptions of these genes. However, *S. meliloti* mutants of two principle genes, *aceA* (encoding isocitrate lyase) and *glcB* (encoding malate synthase), in the glyoxylate cycle were not impaired in nodulation and nitrogen fixation on alfalfa [85]. *NGR_c03920- NGR_c03950* expressed higher in *V. unguiculata* bacteroids (RPKM = 254–401) than in *L. leucocephala* bacteroids (RPKM = 55–105) and the free-living condition (RPKM = 24–40). They encode glycolate oxidase which catalyses the oxidation from glycolate and O_2_ to glyoxylate and H_2_O_2_. The *glcD* (*NGR_c03920* homolog) mutant of *A. caulinodans* formed Fix^-^ nodules on *S. rostrata* [86]. On the other hand, it has been reported, in *S. meliloti-Medicago* symbiosis, an optimal level of H_2_O_2_ is required in the normal progression of infection threads and the efficient release of bacteria into nodule cells [87], [88]. In this study, *NGR_b11000* encoding catalase C (peroxidase) was up-regulated in both *V. unguiculata* bacteroids (RPKM = 19) and *L. leucocephala* bacteroids (RPKM = 48) compared to the free-living form (RPKM = 4). However, the symbiotic roles of glycolate oxidase and products from its activation in bacteroids remain elusive. Despite the unclear picture of the symbiotic role of glyoxylate cycle, there were large amounts of acetate and fatty acids in the nodules [89]. On the other hand, as shown in Table 3S, the down-regulation of fatty acid synthesis genes and the up-regulation of fatty acid metabolism genes in bacteroids might be related to the presence of fatty acids in nodules.

As shown in Figure 1E and 1F, PHB granules were observed in bacteroids of nodules from both *L. leucocephala* and *V. unguiculata*. In line with this observation, the acetyl-CoA acetyltransferase coding gene, *phbA* (*NGR_c32720*, 3-fold) and the poly-beta-hydroxybutyrate polymerase coding gene *phbC1* (*NGR_c34290*, 4- and 7-fold, respectively) were up-regulated in both legume hosts while *phbC2* (*NGR_c14000*) and *phbB* (*NGR_c32710*) were constitutively expressed. Noteworthy, *phbZ* encoding a polyhydroxybutyrate depolymerase (*NGR_b03370*) was up-regulated by 19-fold and 9-fold in *L. leucocephala* and *V. unguiculata*, respectively. *bdhA2* encoding a 3-hydroxybutyrate dehydrogenase (*NGR_c23850*) was constitutively expressed. Therefore, PHB level is subject to strict modulation in bacteroids. It has been reported that PHB was not accumulated in the free-living culture of *R. etli* when biotin was added [90]. The transcription of *bdhA* in free-living *S. meliloti* was higher in the presence of added biotin [91]. These earlier reports suggested biotin-induced PHB degradation in the free-living condition. However, *bioABDF* involved in biotin synthesis were all up-regulated in *L. leucocephala* (3- to 9-fold) but down-regulated in *V. unguiculata* nodules (3-fold), indicating a potential complex regulation on PHB level in bacteroids.

Notably, PHB granules in *L. leucocephala* bacteroids were larger than those in *V. unguiculata* bacteroids (Figure 1E and 1F). Phasins encoded by *phaP* genes could bind to PHB granules and promote PHB synthesis by regulating the surface/volume ratio of PHB granules or by interacting with the polyhydroxyalkanoate synthase [92]. It was also reported that *phaP1* and *phaP2* in *S. meliloti* contributed to PHB accumulation in bacteroids and symbiotic nitrogen fixation [93]. NGR234 has three *phaP* homologs, *phaP1* (*NGR_c03360*), *phaP2* (*NGR_c13240*), *phaP3* (*NGR_a00900*). As shown in Table 4, *phaP3* seemed to be specifically and strongly expressed in bacteroids of both legume hosts while *phaP1* and *phaP2* were up-regulated in *L. leucocephala* bacteroids (1.8-fold) and *phaP1* was down-regulated in *V. unguiculata* bacteroids. Therefore, these expression patterns of *phaP* homologs agree well with the difference in PHB granule size observed in *L. leucocephala* and *V. unguiculata* bacteroids.

**Table 4:** Expression patterns of *phaP* genes encoding phasins.

		phaP3 (408 bp)	phaP1(447 bp)	phaP2 (354 bp)
Unique reads	(F)	2	8114	12203
	(LB)	7063	1693	2592
	(VB)	21683	1271	6715
Coverage	(F)	30.4%	99.8%	99.7%
	(LB)	99.8%	99.8%	99.7%
	(VB)	99.8%	99.8%	99.4%
RPKM	(F)	0.64	2379.38	4518.51
	(LB)	19536.37	4274.29	8263.17
	(VB)	15673.09	838.56	5594.2
Ratio	(LB/F)	30404.78	1.8	1.83
	(VB/F)	24392.3	0.35	1.24
	(LB/VB)	1.25	5.1	1.48
P-value	(LB/F)	0	7.38E-94	2.42E-150
	(VB/F)	0	0	6.1051E-44
	(LB/VB)	4.19E-56	0.00E+00	3.30E-60
FDR	(LB/F)	0	9.25E-93	4.48E-149
	(VB/F)	0	0	2.20E-43
	(LB/VB)	1.59E-54	0	1.35E-58

Note: VB/F, *V. unguiculata* bacteroids vs free-living bacteria; LB/F, *L. leucocephala* bacteroids vs free-living bacteria; LB/VB, *L. leucocephala* bacteroids vs *V. unguiculata* bacteroids; RPKM (reads per kilobase per million mapped reads); FDR (false discovery rate).

### Conclusions

Although *Sinorhizobium* sp. NGR234 is well known for its broad-host range and the ability of forming either determinate or indeterminate nodules on corresponding legumes [24], genome-level transcriptomic adaptions to determinate or indeterminate nodules of different legumes have not been investigated for this strain before. By using RNA-seq, we conducted a high-resolution transcriptomic analysis for NGR234 bacteroids in indeterminate nodules of *L. leucocephala* and in determinate nodules of *V. unguiculata*. Both common and distinct transcription patterns were uncovered for NGR234 bacteroids in these two non-model legumes. These shed new light on the mysterious mechanisms of rhizobial adaptations to diverse legume hosts.

## Supporting Information

Table S1 Transcriptomic profiles of *Sinorhizobium* sp. NGR234. (XLS)

Table S2 Differentially expressed genes of *Sinorhizobium* sp. NGR234 in this study. (XLS)

Table S3 KEGG pathway enrichment of differentially expressed genes. (XLS)

Table S4 qRT-PCR validation of transcriptomic profiles. (XLS)
